# Automatic Detection of Onset and Offset of Respiratory Electromyographic Activity in Severe COPD Patients on Non-Invasive Mechanical Ventilation

**DOI:** 10.1109/JTEHM.2026.3657639

**Published:** 2026-01-23

**Authors:** Abel Torres, Luis Estrada-Petrocelli, Tim Raveling, Marieke L. Duiverman

**Affiliations:** Universitat Politècnica de Catalunya—BarcelonaTech (UPC) Barcelona 08034 Spain; Institute for Bioengineering of Catalonia (IBEC), BIST Barcelona 08028 Spain; Centro de Investigación Biomédica en Red de BioingenieríaBiomateriales y Nanomedicina (CIBER-BBN) Madrid 28029 Spain; Facultad de Ingeniería, Secretaría Nacional de Ciencia, Tecnología e Innovación (SENACYT) Sistema Nacional de Investigación (SNI)Universidad Latina de Panamá Panama City 0823-00933 Panama; Department of Pulmonary Diseases and Home Mechanical VentilationUniversity Medical Center Groningen, University of Groningen Groningen 9713 GZ The Netherlands

**Keywords:** Chronic obstructive pulmonary disease (COPD), non-invasive ventilation (NIV), surface diaphragm electromyography (EMGdi), patient-ventilator asynchrony (PVA), fixed sample entropy (fSE).

## Abstract

Objective: Accurate detection of inspiratory onset and offset in the diaphragm electromyographic signal (EMGdi) is clinically relevant to assess patient-ventilator interaction in COPD patients undergoing non-invasive ventilation (NIV). Manual annotations are time-consuming and subject to inter-observer variability, highlighting the need for reliable automatic methods. Method: We developed a fully automatic algorithm to detect EMGdi activity cycles and their onset/offset timing in overnight NIV recordings. Four ECG suppression approaches were combined with root mean square (RMS) and fixed sample entropy (fSE) envelopes, and a novel bias correction strategy based on inspiratory-to-basal signal-to-noise ratio (I2BSNR) was introduced. Performance was compared with double-blind annotations from two independent experts. Results: In a cohort of 10 severe COPD patients (9212 annotated cycles), the best configuration (adaptive filtering with fSE exponential envelope) achieved F $1=0.96$, with onset bias −28 ms (SD 270 ms) and offset bias + 120 ms (SD 292 ms). We show that fSE-based envelopes consistently outperform RMS in onset/offset detection, and that I2BSNR-based correction reduces systematic bias to within accepted clinical timing windows. Conclusions: The proposed method provides accurate and robust onset/offset detection of EMGdi during NIV in COPD patients. This enables reliable quantification of patient-ventilator asynchronies such as ineffective efforts and delayed cycling, offering direct clinical value for optimizing nightly ventilator settings in severe COPD. Clinical and Impact: Reliable detection of patient inspiratory activity offers a practical tool to guide real-time ventilator adjustments and reduce patient-ventilator asynchronies

## I. Introduction

Chronic obstructive pulmonary disease (COPD) is presently one of the leading causes of morbidity and mortality worldwide, with its position in the global ranking of disease impact projected to rise over the next decade [1]. Respiratory muscle dysfunction is increasingly recognized as a contributor to disease progression and symptom burden in both acute and chronic respiratory failure [2].

Non-invasive ventilation (NIV) constitutes a key therapy to treat acute exacerbations and chronic hypercapnic respiratory failure in COPD, improving gas exchange and health-related quality of life, and reducing mortality and hospital readmissions [3]. Nevertheless, successful NIV hinges on close synchrony between the patient’s own respiratory drive and the pressure or flow assistance delivered by the ventilator. Patient-ventilator asynchrony (PVA) is common in COPD, occurring in more than 25% of breaths in up to one-third of clinical NIV recordings [4], [5]. PVA increases the work of breathing, causes discomfort and sleep disruption, reduces ventilatory support efficiency and has been linked to worse clinical outcomes [5], [6].

Surface electromyography of the diaphragm (EMGdi) provides a real-time, non-invasive view of the neural respiratory drive that triggers inspiratory effort. Standardized electrode placement and signal processing recommendations from the ATS/ERS statement on respiratory muscle testing [7], together with insights from a recent expert round table report on respiratory surface EMG analysis and applications [8], as well as a recent quantitative comparison of respiratory surface EMG electrode configurations [9], have paved the way for wider clinical and research use of EMGdi. By analyzing EMGdi signals, clinicians gain insight into the timing of respiratory events, such as the onset and offset of diaphragmatic contraction, which are essential for assessing patient-ventilator interaction [10]. Accurate detection of these respiratory events can enhance the synchronization of the ventilator with the patient’s spontaneous breathing efforts, thereby improving the overall effectiveness of NIV in COPD patients. Recent cohort and proof-of-concept studies have demonstrated how continuous EMGdi monitoring can quantify neural drive, flag occult PVA, and guide titration of pressure support and positive end-expiratory pressure during hospital and home NIV [4], [11], [12].

However, automated interpretation of surface EMGdi remains challenging. First, the diaphragm signal is typically embedded in a low signal-to-noise ratio environment, being contaminated by motion artefact, power line interference and, most prominently, overlapping cardiac electrical activity [8]. Second, respiration-related changes in EMG amplitude and complexity vary widely both between and within patients as lung mechanics and body position evolve. These issues are exacerbated during home NIV, where frequent changes in body position, unstable electrode contact and cable or interface artefacts further degrade the quality of surface EMGdi recordings and result in even lower signal-to-noise ratios. These factors make surface EMGdi processing during home NIV considerably more challenging and motivate the need for a robust, multi-stage signal processing pipeline capable of operating reliably on long, noisy overnight data. In the present work, we focus specifically on these signal processing and validation aspects, rather than on analyzing clinical outcomes or NIV adjustment protocols.

Traditional amplitude-based algorithms, such as simple root mean square (RMS) envelopes, demand careful threshold tuning. These algorithms misclassify a considerable number of cycles when the ECG/EMGdi ratio is high or breathing becomes erratic [11]. Extensive research has led to a spectrum of ECG interference mitigation techniques (high-pass filtering [13], targeted gating [14], [15], wavelet denoising [16], [17], and adaptive least mean squares (LMS)-based filtering [18], [19], [20], [21]) and to the development of complementary envelope metrics. While RMS remains the most used descriptor of EMG amplitude [12], [15], fixed sample entropy (fSE) provides greater robustness to residual artefacts and low-frequency drift [22], [23]. Building on these advances, recent signal processing approaches combine adaptive or wavelet-based ECG subtraction, multi-feature envelope extraction and cycle-by-cycle threshold adaptation. In parallel, machine learning and deep learning strategies are rapidly emerging to detect and classify PVA events directly from ventilator waveforms or diaphragmatic activity [24]. These data-driven approaches achieve high accuracy in experimental datasets but their transferability to long, noisy home NIV recordings still requires custom feature design and rigorous external validation.

The present study introduces an integrated framework for robust detection of inspiratory activity in EMGdi by combining three complementary methodological advances: (i) a comparative assessment of four ECG suppression strategies (wavelet denoising, targeted gating preceded by a 45 Hz high-pass filter, adaptive filtering, and adaptive filtering followed by gating without the high-pass filter), each independently evaluated for their ability to minimize cardiac interference while preserving inspiratory EMGdi energy; (ii) a dual path envelope analysis that processes RMS- and fSE-based representations in parallel, where in each path three envelopes are assessed (a 250 ms sliding window envelope, the same envelope smoothed with a 300 ms moving average, and the base-10 exponential of that smoothed envelope); and (iii) a bias correction model that compensates for systematic onset/offset timing shifts as a function of signal quality, quantified by the inspiratory-to-basal signal-to-noise ratio (I2BSNR). Using an annotated overnight database of COPD patients on home NIV, we perform a methodological validation of the proposed framework, quantifying event-detection performance and onset/offset timing errors against expert visual annotations. The clinical application of this pipeline to guide NIV setting adjustments and to evaluate its impact on patient outcomes is left for future studies.

## II. Materials

### A. Signal Acquisition

The EMGdi signals were acquired using two disposable 24 mm surface electrodes (Arbo^TM^ H124SG, Ref. Code: 31.1245.21, Kendall^TM^, Covidien, Germany) placed bilaterally midclavicular at the costal margin, with a common electrode placed on the sternum. Additionally, a pressure sensor was positioned between the mask and the ventilator to measure the pressure changes applied during ventilation. Both signals were amplified and analogue pre-filtered between 0 and 200 Hz, and digitized for further processing and analysis using a data acquisition system (Dipha-16®, Demcon, Macawi Respiratory Systems, the Netherlands) with a sample frequency of 500 Hz.

### B. Dataset and Ethics

The dataset employed in this study comprises EMGdi signals obtained from patients with severe COPD undergoing home NIV, utilizing one hour extracted from the overnight recording. This signal database was acquired at the Department of Pulmonary Diseases within the University Medical Center Groningen in the Netherlands, with approval from the Medical Ethical Committee of the University Medical Center Groningen (ClinicalTrials.gov NCT02652559 and NCT03053973). All patients provided written informed consent for participation in the trial. The demographic and functional characteristics of the analyzed patients are summarized in Table 1, and the ventilation settings and synchrony parameters are presented in Table 2.

**TABLE 1:** Patient Characteristics

COPD Patient	Sex (F/M)	Age (years)	BMI (kg/m ${}^{2}$)	FEV1 (L)	FEV1/FVC (%)	RV/TLC (%)
P1	M	62	26	0.68	23	61
P2	F	71	30	0.75	35	61
P3	F	59	23	0.59	22	63
P4	M	63	22	0.97	23	61
P5	F	57	22	0.33	22	86
P6	F	73	25	0.56	43	74
P7	M	73	17	0.59	26	73
P8	M	81	35	1.09	35	49
P9	M	70	29	0.74	29	66
P10	M	47	28	0.58	24	59
Sum/Avg	4/6	65.6	25.7	0.69	28.2	65.3

BMI: body mass index; FEV1 (L): forced expiratory volume in 1 second; FEV1/FVC (%): ratio of forced expiratory volume in 1 second to forced vital capacity; RV/TLC (%): ratio of residual volume to total lung capacity.

**TABLE 2:** Ventilation Settings and Synchrony Parameters

COPD Patient	VM (PC/ST)	IPAP (cmH_2_O)	EPAP (cmH_2_O)	BURR (b/min)	IT (PC) / ITmax (ST)
P1	ST	27	5	14	1.0
P2	ST	28	5	14	1.0
P3	ST	25	6	12	1.0
P4	ST	32	4	22	0.9
P5	ST	23	6	17	1.0
P6	PC	23	4	14	1.0
P7	PC	22	5	14	1.0
P8	ST	20	6	16	1.3
P9	PC	22	4	14	1.0
P10	PC	26	6	14	1.0
Sum/Avg	6/4	24.8	5.1	15.1	1.02

VM: Non-invasive ventilation mode; PC: pressure control; ST: spontaneous/timed; IPAP: inspiratory positive airway pressure; EPAP: expiratory positive airway pressure; BURR: backup respiratory rate; IT(PC)/ITmax (ST): set inspiratory time for PC modes or maximum inspiratory time limit for ST modes (seconds).

### C. Expert Annotation Protocol

Two experienced clinicians independently annotated EMGdi onset and offset using a custom graphical interface that simultaneously displayed the EMGdi signal filtered between 5 and 200 Hz, together with a 50 Hz high-pass filtered EMGdi trace (which attenuated part of the EMGdi amplitude but removed most ECG interference, improving the visibility of the diaphragmatic activation), and the airway pressure signal recorded by the ventilator. The graphical annotation interface was developed in Matlab® (The Mathworks, Inc., R2023a, Natick, MA, USA) and used solely for signal visualization during manual labeling. EMGdi onset and offset were always defined from the EMGdi traces, while pressure was inspected only as contextual information and was not used to set the reference timing. No RMS, fSE or other derived envelopes were used during annotation. Both experts were experienced clinicians familiar with NIV waveform interpretation and surface EMGdi analysis. Before the formal scoring, they jointly reviewed a set of representative examples using the same graphical interface that was later used for annotation. Onset and offset were marked as the beginning and end of visible EMGdi activity rising above basal expiratory levels. In particular, the experts defined EMGdi offset as the end of the main inspiratory burst, i.e., the point at which the EMGdi activity returned to basal expiratory levels, corresponding to the neural end-inspiratory time that they routinely use in clinical practice to assess patient-ventilator cycling asynchronies in NIV. The scoring assessments were conducted in a blinded fashion, with each scorer unaware of the annotations made by the other. Synchrony between the annotations of the two experts was assessed on a cycle-by-cycle basis by comparing the timestamps of their inspiratory onset and offset markers. Each scorer annotated inspiratory EMGdi activity as onset-offset intervals. Intervals from both scorers that overlapped in time were considered to refer to the same inspiratory effort and were paired, whereas intervals without overlap were treated as EMGdi activations identified only by one scorer.

### D. Definition of Reliable Events

For the assessment of automatic detection of EMGdi activity, the automatic detector was applied to the full one-hour segment of each recording, and only reliable events were considered as reference, defined as inspiratory cycles for which both scorers annotated overlapping inspiratory EMGdi activity. Cycles annotated by only one scorer or without a unique match between scorers were excluded from the event level analysis. Event level performance was evaluated using sensitivity, precision and F1-score derived from true positives (TP), false negatives (FN) and false positives (FP). An automatically detected inspiratory cycle was counted as a TP when it overlapped in time with a reliable event, whereas a reliable event with no overlapping automatic detection was counted as an FN. Events classified as unreliable were excluded from the reference set: automatic detections overlapping only these unreliable events were not included in the TP/FP/FN counts. This definition of reliable events was based solely on inter-scorer agreement and not on visual signal quality or signal-to-noise ratio.

For onset and offset timing, a tolerance value of 150 ms was used to ensure reliable inter-scorer agreement. This threshold was defined by the clinical experts as the maximum discrepancy compatible with normal visual variability when identifying EMGdi onset and offset in long, noisy NIV recordings [4], [5], [8], [11]. Its purpose was solely to ensure reference reliability by excluding events with substantial inter-scorer disagreement. Onset and offset were treated as independent events (exclusion of one marker did not automatically invalidate the other). While most events showed excellent agreement between the two clinicians, occasional discrepancies occurred, particularly when EMGdi activation overlapped with an ECG complex or when activation was very low in amplitude. For each onset of offset that satisfied this 150 ms criterion, the reference time used to evaluate the automatic detectors was taken as the arithmetic mean of the two expert markers. Table 3 presents the total EMGdi events annotated by each scorer, the total number of reliable EMGdi events (with annotations from both scorers), the total number of reliable onsets where the difference between scorers was less than 150 ms, the root mean square difference (RMSD) between scorers for reliable onsets, and the number and RMSD of reliable offsets. This explains why the number of onsets and offsets examined is not identical. The automatic detection of 9212 EMGdi events was analyzed in total, with the automatic detection of onset and offset being examined in 6625 and 6498 of these events, respectively.

**TABLE 3:** Summary of Expert Annotations of EMGdi Events

COPD Patient	Scorer1 Events	Scorer2 Events	Reliable Events	Reliable Onsets	RMSD Onsets (ms)	Reliable Offsets	RMSD Offsets (ms)
P1	994	970	964	610	77	567	81
P2	1254	1222	1218	719	80	725	79
P3	889	875	871	656	68	607	78
P4	1168	829	807	517	77	486	82
P5	636	636	583	416	75	383	83
P6	822	903	821	630	70	525	80
P7	1088	1121	1067	629	71	838	74
P8	812	848	754	411	82	414	83
P9	1363	1366	1353	1280	65	1294	58
P10	798	777	774	757	49	659	63
Sum/Avg	9824	9547	9212	6625	71.4	6498	76.1

VM: Non-invasive ventilation mode; PC: pressure control; ST: spontaneous/timed; IPAP: inspiratory positive airway pressure; EPAP: expiratory positive airway pressure; BURR: backup respiratory rate; IT(PC)/ITmax (ST): set inspiratory time for PC modes or maximum inspiratory time limit for ST modes (seconds).

## III. Methods

### A. EMGdi Signal Preprocessing

**FIGURE 1.**
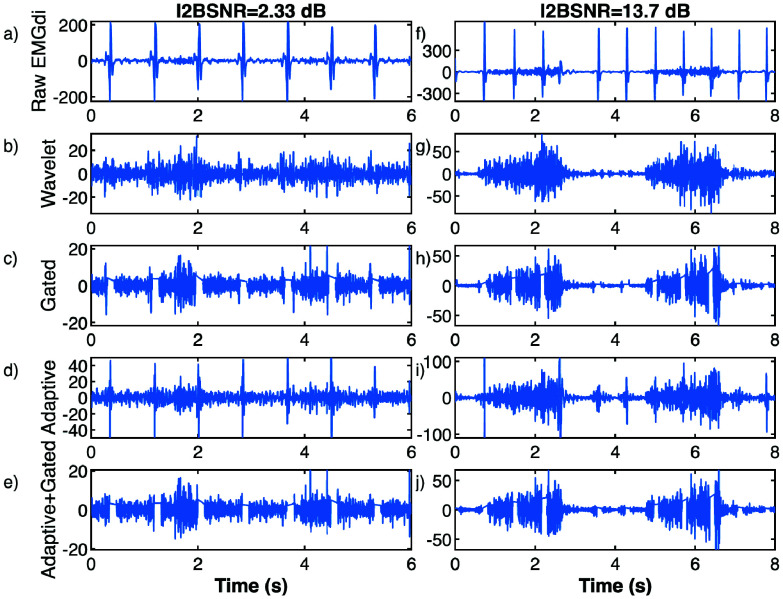
Example of ECG removal methods applied over two EMGdi signals, one with a low I2BSNR (a, b, c, d and e), and other with high I2BSNR (f, g, h, i and j). (a and f) Preprocessed EMGdi signals. (b and g) EMGdi signals after adaptive wavelet thresholding, (c and h) EMGdi signals post-gating, (d and i) EMGdi signals after adaptive filtering, and (e and j), adaptively filtered signals with gating applied.

EMGdi signals were filtered with a 4th-order zero-phase Butterworth band-pass filter between 5 and 200 Hz, consistent with the analogue 0-200 Hz bandwidth of the acquisition system, to remove slow baseline drift and high-frequency noise within the available band. Power line interference at 50 Hz and its harmonics were attenuated using adaptive narrowband zero-phase IIR notch filters, whose bandwidth (Q-factor) was iteratively adjusted so that the residual line power at each mains harmonic matched the average power in adjacent sidebands. R-peaks were then automatically detected using a QRS detector based on the Pan-Tompkins algorithm [23] applied to the EMGdi signal filtered between 5 and 40 Hz, and all samples within a window from 135 ms before to 365 ms after each R-peak were excluded to obtain segments free of cardiac complexes. For each recording, consecutive 30 s epochs were analyzed. Within each epoch inspiratory segments were obtained by concatenating the samples belonging to EMGdi inspiratory intervals (between onset and offset marks) that lay outside the ECG exclusion windows, whereas basal expiratory segments were obtained from the remaining samples outside both inspiratory intervals and ECG exclusion windows. The inspiratory-to-basal signal-to-noise ratio (I2BSNR) was then calculated as the ratio of root mean square (RMS) values between these inspiratory and the basal expiratory segments, expressed in decibels (dB). Fig. 1 shows examples of two preprocessed EMGdi signals: (a) shows a signal with low I2BSNR while (f) shows a signal with high I2BSNR.

### B. ECG Removal

#### 1) Wavelet Denoising With Adaptive Thresholds

The wavelet-based adaptive filtering algorithm applied in this work for ECG denoising is inspired by the methodology proposed by Zhan et al. [16]. This algorithm is based on discrete wavelet decomposition followed by signal reconstruction using adaptive thresholds applied to approximation and detail coefficients. Specifically, we employ a Daubechies 4 wavelet and perform decomposition up to level 4 to effectively filter out noise while preserving the EMGdi signal’s essential features. This level of decomposition, utilizing a sampling frequency of 500 Hz, ensures that at the fourth level of decomposition, the approximation coefficients contain information from signals with frequencies lower than 15.625 Hz (primarily corresponding to P-T waves cardiac components). Consequently, in the wavelet reconstruction of the EMGdi signal, only the detail coefficients subjected to the adaptive threshold proposed by Zhan et al. [16] are utilized. In this algorithm, the lower and upper bounds of the threshold window, denoted as *Lb* and *Ub* respectively, are set to 200 and 300 ms, and the parameter $K$ is fixed at a value of 5. In Fig. 1 (b) and (g), examples of the two EMGdi signals filtered using this ECG component removal method are depicted.

#### 2) Gating

Gating is a method aimed at removing segments of the EMGdi signal containing cardiac activity [25]. To apply this technique, R-peak detection was initially performed using a QRS complex detection algorithm based on the Pan-Tompkins algorithm [26]. The R-peak detection algorithm was applied to the EMGdi signal, which was filtered between 5 and 40 Hz using a zero-phase fourth-order Butterworth filter to enhance the cardiac components of the signal. Subsequently, EMGdi signal samples from a window centered on the R-peak detection were replaced by a line connecting the RMS of the EMGdi signal in windows adjacent to the window centered on the R-peak. However, when applying this technique to the original EMGdi signal, the percentage of signal samples requiring replacement to eliminate cardiac activity would be very high, especially at high heart rates, as it necessitates the removal of samples corresponding to the entire PQRST complex. Therefore, it is necessary to apply prior high-pass filtering to remove most of the energy from the low frequency cardiac components (P and T waves), leaving only the higher frequency activity corresponding to the QRS complex. In this study, a zero-phase second-order Butterworth filter with a cutoff frequency of 45 Hz was applied. Once filtering was applied, to remove the remaining cardiac activity, only the samples corresponding to a 130 ms window centered on the detected R-peaks needed replacement. For each R-peak, the removed samples were reconstructed as a straight line joining the RMS value of the EMGdi signal computed over a 65 ms window immediately preceding the gated segment and the RMS value computed over a 65 ms window immediately following it. Examples of the two EMGdi signals with high and low I2BSNR after applying the gating technique are shown in Fig. 1 c) and h), respectively.

#### 3) Adaptive Filtering

An adaptive noise cancellation technique based on event-synchronous cancellation can effectively mitigate ECG interference in recorded EMGdi activity [19], [20]. This adaptive noise cancellation scheme requires a reference input that is correlated with the ECG interference in the EMGdi signal but uncorrelated with EMG activity. Because the signal database from the clinical trials did not include a separate ECG channel, this reference input was synthesized from the EMGdi signal itself, following previously described procedures [19]. To generate this reference input, R-peak detections are utilized to create a template by averaging EMGdi signal segments within a 600 ms time window, starting 200 ms prior to the R-peak and ending 400 ms after the R-peak. The 25% of segments with the highest energy (showing higher EMGdi activity) were excluded before averaging. This template is then replicated at each R-peak location to form a periodic beat train, which is used as the reference input to the adaptive filter, while the original EMGdi signal acts as the main input. The adaptive algorithm employed was implemented using a least mean squares (LMS) algorithm, with the number of weights of the linear combiner corresponding to 0.4 seconds of data and an adaptation constant adjusted so that the cancellation signal energy matches that of the reference signal. Examples of this adaptive filter applied to signals with high and low I2BSNR are shown in Fig. 1 (d) and (i). In addition, the gating technique was applied to the adaptively filtered signal. The resulting adaptive-plus-gating signals are illustrated in Fig. 1 (e) and (j).

### C. EMGdi Envelope Signal

#### 1) Root Mean Square Envelope

**FIGURE 2.**
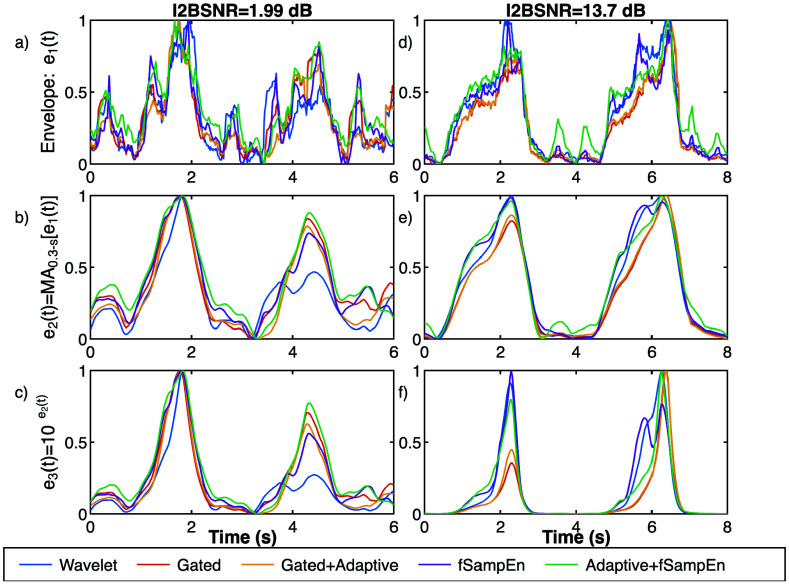
Example of EMGdi envelopes applied over two EMGdi signals, one with a low I2BSNR (a, b and c), and other with high I2BSNR (d, e, and f); (a and d) first RMS or fSE envelope (e1) applied on 250 ms windows, (b and e) second envelope (e2) with a 300 ms moving average of e1, and (c and f) third envelope (e3) calculated as $10^{\mathrm {e2}}$.

To detect electromyographic activity in the denoised EMGdi signals, three envelopes based on RMS filters were analyzed. The first envelope (e1) was obtained by directly calculating the RMS over a 250 ms moving window. To reduce false onset or offset detections caused by noise or residual ECG activity, the e1 envelope was smoothed with a 300 ms moving average filter, yielding the second envelope (e2). Finally, to enhance high-amplitude variations and attenuate low-amplitude variations, the e2 envelope was used as an exponent for base 10 to create the third envelope (e3). Fig. 2 shows examples of these three envelopes applied to the denoised EMGdi signals with high and low I2BSNR used in Fig. 1. To facilitate comparison of all envelopes, Min-Max normalization has been applied.

#### 2) Fixed Sample Entropy Envelope

Fixed sample entropy [22] is a technique that has been used in the study of respiratory muscle EMG recordings in healthy subjects and in patients with COPD at different levels of inspiratory load [11], [23], [27], [28], [29], [30]. In these studies, the performance of fSE has been shown to be superior to other techniques used in clinical practice for the analysis of respiratory EMG signal activity. The advantage of this method is its sensitivity to variations in amplitude and complexity of the random components of the EMG activity while remaining unaffected by changes in amplitude of non-random components (such as ECG activity, which exhibits a deterministic and repetitive pattern). Thus, fSE can be applied to the EMGdi signal without employing any ECG removal technique. To compute the envelope of the EMGdi signal using the fSE algorithm, an embedding dimension $m=1$, a tolerance value $r$ equal to 0.4 times the standard deviation of the preprocessed EMGdi signal, and the same 250 ms moving window as that used for calculating the first envelope based on RMS were applied. Furthermore, starting from this initial envelope based on fSE, the same procedure as in RMS was followed to obtain envelopes e2 and e3. Fig. 2 depicts examples of the envelope signals calculated with fSE. Additionally, fSE was applied to the EMGdi signal after adaptive filtering. However, applying fSE to signals processed with the wavelet denoising or gating is not suitable, as these techniques distort the random components of the EMGdi signal, producing a notch or downward shift in fSE values.

### D. EMGdi Event Detection

**FIGURE 3.**
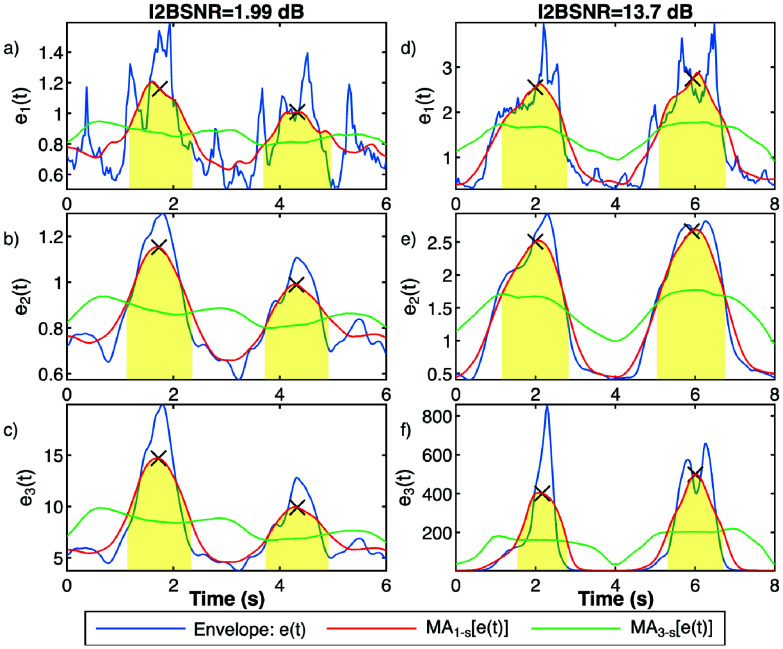
Illustration of respiratory EMGdi activity detection using fSE envelopes (e1, e2 and e3) applied to two EMGdi signals: one with a low I2BSNR (a, b, c), and another with high I2BSNR(d, e, f). Envelopes are shown in blue curves, the 1 s moving average shown in red, the 3 s moving average in green, and detection markers indicated by crosses.

To detect respiratory EMGdi activity, we employ two moving averages: a low-frequency moving average with a 3 s window to capture slow baseline changes in EMGdi activity, largely smoothing out respiratory fluctuations, and a high-frequency moving average with a 1 s window that attenuates cardiac-related variations while preserving the respiratory component. EMGdi event detection is annotated at the midpoint between crossings of these two moving averages. For each detected event, we then compared the mean value of the EMGdi envelope within the inspiratory interval with the mean envelope amplitude in two reference windows, each covering 20% of the cycle duration immediately before onset and immediately after offset. Cycles with durations less than 200 ms and cycles in which the mean inspiratory amplitude was less than 10% higher than the mean amplitude in these surrounding windows were excluded from further analysis, to discard events with essentially flat envelopes and likely false detections or artefacts rather than genuine inspiratory efforts. Fig. 3 illustrates an example of these moving averages applied to envelopes e1, e2, and e3 computed using fSE for signals with high and low I2BSNR, detecting two EMGdi events.

### E. Detection of EMGdi Onset and Offset

**FIGURE 4.**
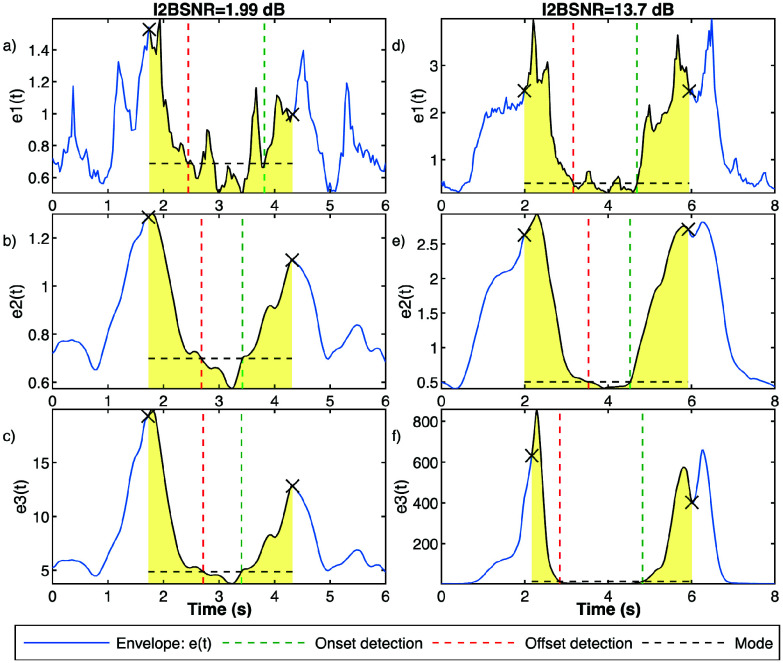
Illustration of respiratory EMGdi activity onset and offset detection applied to fSE e1, e2 and e3 envelopes applied to two EMGdi signals, one with a low I2BSNR (a, b and c), and other with high I2BSNR (d, e, and f). Envelopes are represented by blue curves, cycle detection markers by black dots, the mode between cycle marks is indicated by a black dashed line, and onset and offset detections are depicted by red and green dashed lines, respectively.

Once cycles are detected, EMGdi activity onset and offset are identified based on adaptive thresholds calculated using preceding and succeeding activity around the analyzed cycle [11]. Kernel density estimation algorithm [23] is employed to determine onset and offset thresholds by computing the mode of the envelope between the current cycle and its adjacent cycles. After threshold computation, starting from the cycle detection mark, the mark is adjusted backward and forward until these thresholds are crossed. Cycle onset and offset are defined as the last envelope sample exceeding these thresholds. Cycles with an onset-to-offset difference of less than 200 ms were excluded. Additionally, we compute the Shannon entropy of the probability distribution of the envelope during the phase between onset and offset. This entropy is then compared with the entropy calculated in preceding and succeeding phases, between offset and onset. If the entropy during the cycle is lower than in any of the adjacent phases, the cycle is eliminated from further analysis. Fig. 4 illustrates an example of the mode calculated between two consecutive detected cycles and the corresponding onset and offset detections for EMGdi signals with low and high I2BSNR.

### F. Signal Quality Based Correction

To evaluate the influence of the I2BSNR on envelope calculation and the onset/offset detection algorithm of the EMGdi activity, 89 signals were simulated with RMS ratios between inspiratory and basal segments ranging from 1.2 to 10 (corresponding to I2BSNR levels of 1.58-20 dB). Each signal consisted of 2000 respiratory cycles. The stochastic EMGdi component was simulated using Gaussian white noise, and the amplitude of the inspiratory segment was increased according to the following function:

\begin{align*} e\left ({{ t }}\right)={\begin{cases} 1-4\left ({{ t-1 }}\right)^{2}~\mathrm {if}~0.5 \le t\le 1.5 \\ 0~\mathrm {otherwise} \\ \end{cases}} \tag {1}\end{align*}

**FIGURE 5.**
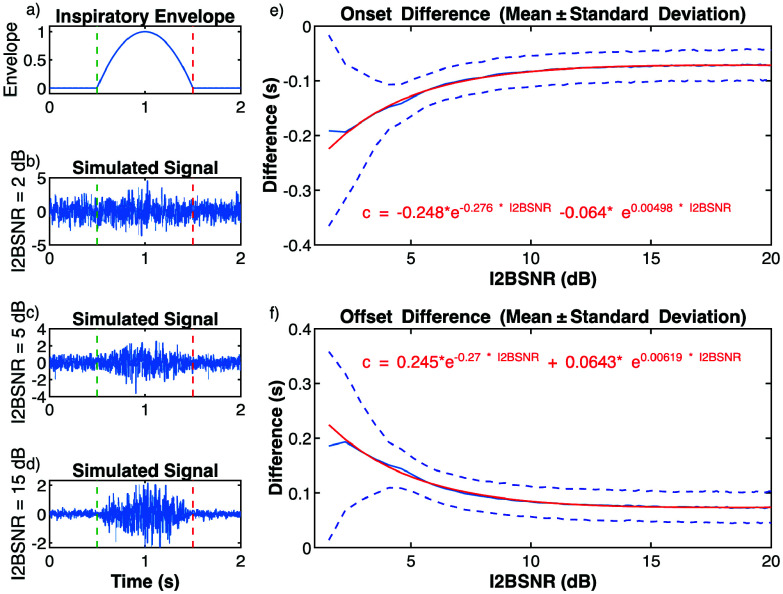
(a) Inspiratory envelope. (b-d) Simulated signals with I2BSNR values of 2, 5, and 15 dB, respectively. (e) Mean ± standard deviation of the difference between onsets, and (f) offsets, with their respective exponential fits for the third envelope calculated using fSE.

Fig. 5 (a), (b), (c), and (d) illustrate an example of the simulated inspiratory signal envelope, along with simulated EMGdi signals with I2BSNR values of 2, 5, and 15 dB, respectively. After generating the simulated EMGdi signals, the envelopes were calculated, the cycle detection algorithm was applied, and the onset and offset detection algorithms were implemented. For each noise level, the mean and standard deviation (SD) of the difference between the detected onsets and offsets and the actual onsets and offsets of the simulated envelope were calculated. To analyze the effect of I2BSNR on the mean value of this difference, a fitting process was utilized, employing an equation with two exponential terms to generate a correction function for the onsets and offsets:

\begin{equation*} c\left ({{ I2BSNR }}\right)=a~e^{b~I2BSNR}+c~\mathrm { } e^{d~I2BSNR} \tag {2}\end{equation*}

Fig. 5 (e) and (f) present as an example the mean ± SD of the difference between onsets and offsets, respectively, along with the exponential fit for the e3 case calculated using fSE.

## IV. Results

### A. EMGdi Event Detection Algorithm

Table 4 presents the results of applying the EMGdi event detection algorithm for the five envelope calculation methods and the three types of envelopes used. Among all methods, the third type of envelope demonstrated the best performance in terms of sensitivity and F1-Score. The method based on calculating fSE of the signal, which employs adaptive filtering and uses the third type of envelope, showed the highest sensitivity and F1-Score, detecting 97% of the respiratory diaphragmatic contractions annotated by experts (missing only 239 cycles out of the 9212 events annotated by both experts).

**TABLE 4:** Performance of Automatic EMGdi Event Detection Methods

Method (Envelope)	ED	TP	FN	FP	Se	Pr	F1
Wavelet (e1)	6661	6285	2927	376	0.68	0.94	0.79
Wavelet (e2)	7723	7251	1961	472	0.79	0.94	0.86
Wavelet (e3)	8557	7732	1480	825	0.84	0.90	0.87
Gated (e1)	7833	7207	2005	626	0.78	0.92	0.85
Gated (e2)	7886	7226	1986	660	0.78	0.92	0.85
Gated (e3)	8717	7982	1230	735	0.87	0.92	0.89
Gated+Adap (e1)	8153	7804	1408	349	0.85	0.96	0.90
Gated+Adap (e2)	8786	8369	843	417	0.91	0.95	0.93
Gated+Adap (e3)	8884	8141	1071	743	0.88	0.92	0.90
fSE (e1)	8341	7945	1267	396	0.86	0.95	0.91
fSE (e2)	8319	7995	1217	324	0.87	0.96	0.91
fSE (e3)	9524	8949	263	575	0.97	0.94	0.96
Adaptive+fSE (e1)	8196	7924	1288	272	0.86	0.97	0.91
Adaptive+fSE (e2)	8262	8068	1144	194	0.88	0.98	0.92
Adaptive+fSE (e3)	9392	8973	239	419	0.97	0.96	0.96

ED: Total number of events detected using the corresponding method. TP: Number of true EMGdi events detected by the method. FN: Number of EMGdi events not detected by the method. FP: Number of events detected by the method that are not EMGdi events. Se: Sensitivity=TP/(TP+FN). Pr: Precision=TP/(TP+FP). F1: F1-Score $= 2\ast $Se $\ast $Pr / (Se + Pr).

### B. Evaluation of the EMGdi Onset and Offset Detectors

Tables 5 and 6 present the results of applying the onset and offset detection algorithm, respectively. The number of reliable onsets for each method varies since only the cycles automatically detected by the method (the true positives in Table 4) and where the difference between scorers is less than 150 ms are considered. Before applying the correction based on the I2BSNR, envelope e1 yields the smallest mean difference (MD) for both onsets and offsets, and the smallest root mean squared error (RMSE) for onsets. For offsets, envelope e1 yields the smallest RMSE for non-fSE methods, whereas for fSE-based methods the smallest RMSE is obtained with envelope e3. All MD, RMSE, and SD values are higher for offsets than for onsets.

**TABLE 5:** Performance of automatic EMGdi onset detection methods

Method (Envelope)	RO	Without Correction			With Correction		
		MD	SD	RMSE	MD	SD	RMSE
Wavelet (el)	4630	-130	306	332	-60	305	311
Wavelet (e2)	5322	-326	306	447	-122	308	331
Wavelet (e3)	5500	217	520	563	387	520	648
Gated (e1)	5354	-122	312	335	-52	312	316
Gated (e2)	5335	-268	308	409	-61	312	317
Gated (e3)	5686	156	540	562	328	540	631
Gated+Adap (e1)	5749	-139	256	292	-69	256	265
Gated+Adap (e2)	6057	-296	279	407	-89	281	294
Gated+Adap (e3)	5806	145	542	561	318	540	627
fSE (e1)	5870	-119	312	334	-51	311	316
fSE (e2)	5876	-276	275	389	-73	277	287
fSE (e3)	6450	-196	269	333	-30	269	270
Adaptive+fSE (e1)	5792	-129	287	315	-60	287	293
Adaptive+fSE (e2)	5881	-274	271	385	-72	274	283
Adaptive+fSE (e3)	6465	-193	270	332	-28	270	271

RO: Reliable Onsets (number of EMGdi events detected with the corresponding method where the onset difference between scorers was less than 150 ms). MD, SD, RMSE (in ms): mean difference, standard deviation and root mean square error between the onset automatic onset detection and the average scorer reference, before and after applying the bias correction based on signal quality.

**TABLE 6:** Performance of automatic EMGdi offset detection methods

Method (Envelope)	RO	Without correction			With correction		
		MD	SD	RMSE	MD	SD	RMSE
Wavelet (e1)	4482	348	856	924	277	857	900
Wavelet (e2)	5191	475	514	700	271	516	583
Wavelet (e3)	5484	518	2000	2065	348	2000	2030
Gated (e1)	5270	275	517	585	204	517	556
Gated (e2)	5253	421	496	651	213	498	542
Gated (e3)	5649	488	1801	1866	314	1801	1828
Gated-Adap (e1)	5658	244	367	440	173	366	404
Gated-Adap (e2)	5960	458	416	619	251	417	487
Gated-Adap (e3)	5757	428	1651	1706	255	1652	1671
fSE (e1)	5719	246	428	494	176	427	462
fSE (e2)	5747	443	463	641	240	464	522
fSE (e3)	6336	283	311	420	115	312	332
Adaptive-fSE (e1)	5743	246	397	467	176	397	434
Adaptive-fSE (e2)	5762	449	435	625	248	436	502
Adaptive-fSE (e3)	6348	286	291	408	120	292	315

Definitions as in Table V, with all references to “onset” replaced by “offset”.

### C. Signal Quality Based Correction

**FIGURE 6.**
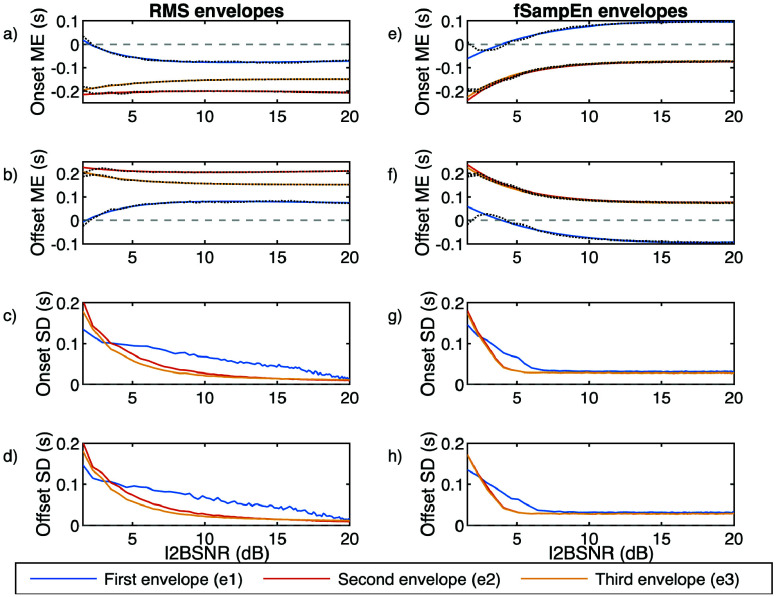
Mean Error (ME) and Standard Deviation (SD) of the differences between detected onsets and offsets using the three types of envelopes with RMS and fSE, and the actual onsets and offsets of the simulated envelope. Plots (a, b, e, f) show the exponential fit of the ME (with actual values in dashed lines). Plots(c, d, g, h) show the SD. Plots (a, b, c, d) present the results for envelopes calculated using RMS. Plots (e, f, g, h) present the results for envelopes calculated using fSE.

Fig. 6 shows the mean error (ME) and SD of the differences between detected onsets and offsets, applying the algorithms to the simulated EMGdi signals, based on the envelopes calculated with RMS and fSE, and the actual onsets and offsets of the simulated envelope. The plots of the mean values display the results of the exponential fit. Generally, the onset detections obtained with the envelopes precede the onsets of the simulated envelope, while the offset detections follow the offsets of the simulated envelope. For RMS, the first envelope presents the smallest ME but shows greater change with increasing I2BSNR. Envelope e2 has an almost constant ME around 0.2 s in absolute value. Envelope e3 starts around 0.2 s and decreases exponentially with increasing I2BSNR, reaching approximately 0.15 s. The SD decreases with I2BSNR, linearly for e1 and exponentially for e2 and e3, with e3 having the smallest SD. For fSE, all three envelopes exhibit exponential variation with increasing I2BSNR. At high SNR levels, e1 changes sign (the envelope detection is delayed compared to the theoretical value). The behavior of e2 and e3 is almost identical in both ME and SD.

The SD for all three envelopes decreases faster than in the RMS case, but the final value reached is higher.

The last three columns of Tables 5 and 6 present the results of applying the correction algorithm based on the I2BSNR. The adjustment of the onset and offset detections was performed for each 30 s segment of the signal, according to the corresponding adjustment equation and the estimated I2BSNR in that segment. The EMGdi signals of the analyzed subjects exhibited an average I2BSNR ranging from 1.97 to 15 dB, with a mean value of 6.02 dB and a SD of 3.78 dB. The SD of the I2BSNR within all analyzed segments for each subject varied between 0.78 and 2.27 dB, with a mean value of 1.27 dB and a SD of 0.42 dB. Using the algorithm to correct onsets and offsets based on the I2BSNR of the signal, the methods that achieve lower ME, SD, and RMSE are those based on fSE using envelope e3.

## V. Discussion

### A. ECG Removal Technique

The goal of ECG removal is to remove cardiac activity from the EMGdi signal while preserving as much respiratory EMG activity as possible. Our investigation focused on four approaches: adaptive filtering, adaptive filtering with gating, wavelet thresholding, and gating techniques, each offering distinct advantages and limitations. These techniques have been extensively studied and implemented in various applications due to their efficacy in managing ECG interference while preserving respiratory EMG activity [8].

The gating technique demonstrated robustness in removing cardiac interference by employing a high-pass filter below 45 Hz. However, this method resulted in a substantial loss of respiratory EMG information within a 130 ms window around QRS complexes, as well as information below 45 Hz, compromising its suitability for applications requiring precise temporal resolution of respiratory events.

The adaptive wavelet denoising technique showed efficacy in retaining respiratory EMG energy by applying non-linear filtering to detail coefficients above adaptive thresholds. Although this approach successfully preserved respiratory EMG activity, it incurred information loss at the lowest approximation level, potentially impacting signal fidelity in lower-frequency ranges (for 500 Hz sampling rate and four decomposition levels, this approximation level corresponds to frequencies below 15.625 Hz). Furthermore, this technique involves thresholding at each decomposition level, resulting in less loss compared to gating, which completely removes EMGdi activity around QRS complexes, but still affecting some EMGdi signal information around QRS complexes.

The adaptive technique demonstrated promising results in preserving respiratory EMG activity by selectively filtering out cardiac-correlated signals using an adaptive filter referenced to a pure cardiac signal. However, it exhibited challenges in eliminating cardiac activity, often leaving residual high-frequency components associated with the QRS complex. To eliminate these residual QRS peaks that appeared in some beats, this technique had to be applied in combination with gating. Therefore, combining adaptive filtering with gating (without the prior high-pass filtering at 45 Hz, which is replaced by this adaptive filtering) addressed this issue, although it came at the expense of sacrificing respiratory EMG information around QRS complexes, limiting its practical utility for onset and offset detections.

In summary, each technique offers a trade-off between ECG interference removal and preservation of respiratory EMG activity. None of them leaves the EMG activity around the QRS complexes unaltered, which may affect the detection of EMG onsets and offsets. The adaptive technique excels at retaining respiratory EMG energy but requires iterative adjustment and either the acquisition of a simultaneous ECG channel or, when this is not available, as in our database, the generation of an ECG-like reference signal derived from the EMGdi. The adaptive wavelet approach provides a balance between noise removal and signal preservation and applies a nonlinear, peak-targeted truncation of wavelet coefficients around ECG complexes, which enables suppression of ECG components that cannot be fully separated using purely linear filtering. This truncation may also distort EMGdi components that overlap ECG in those time-scale regions and sacrifices information from the approximation coefficients (below 15.625 Hz). Conversely, the gating technique efficiently eliminates ECG interference after a 45-Hz high-pass filter, but this comes at the cost of reducing overall EMGdi energy (below 45 Hz) and potentially masking genuine amplitude changes within the windows where gating is applied around the QRS complexes. In our study, for automatic detection of EMGdi onset and offset in COPD patients undergoing home NIV, the adaptive filtering with gating configuration showed the lowest onset and offset timing errors (RMSE) compared with gating alone and wavelet thresholding when all three were combined with the same envelope-based detector. These differences refer specifically to onset/offset detection and do not necessarily reflect the optimal choice for estimating overall EMGdi activity.

### B. EMGdi Envelopes Vs EMGdi Event Detection

In most studies where the envelope of the EMGdi signal is computed, the RMS is directly applied using a moving window [9], [12], [15], [31]. The length of this moving window varies depending on the application; however, if precise detection of onsets and offsets is required, it should be relatively short [8]. In this study, based on prior work [11], [23], a window size of 250 ms was used, which is comparable to the envelopes employed by other research groups (e.g., 200 ms in [15], [31], and 250 ms in [9], [12]). In our case, the envelope was applied to the EMGdi signal after employing one of several ECG removal techniques, including: (1) adaptive wavelet denoising, (2) gating, and (3) adaptive filtering with gating.

In addition to the RMS-based envelopes, an envelope using the fSE was calculated with the same window length. The fSE is a parameter that is relatively insensitive to deterministic activity introduced by the ECG. Therefore, it does not require prior filtering, preserving the relevant EMG activity, which is random in nature. This technique was only applied to the original signal and to the adaptively filtered signal, but not to signals processed by gating or wavelet denoising, since these methods remove EMG segments around QRS complexes, artificially lowering signal complexity and producing spurious notches in the fSE envelope that would bias onset/offset detection.

Among the three RMS-based envelopes and the two fSE-based envelopes, the latter ones exhibited the highest F1-score for respiratory EMGdi event detection (0.91 versus 0.79, 0.85, and 0.90 for RMS-based envelopes using wavelet denoising, gating, and adaptive filtering with gating, respectively). Given that the fSE-based envelope does not require any preprocessing of the signal, this technique presents clear advantages over the other methods.

Due to the low signal-to-noise ratio (EMG activity versus ECG activity), none of the five envelopes analyzed completely attenuated the ECG interference. To minimize its effect on the event detection algorithm, two modifications were applied to the original envelopes: (1) a moving average filter with a duration approximately equivalent to the QRS complex (0.3 s), and (2) an exponential transformation with base 10 applied to the modified envelope. This second transformation markedly enhances higher amplitude variations, in contrast to the lower amplitude variations produced by the QRS complexes. With these two modifications, the F1-scores of the fSE-based techniques increased to 0.96.

### C. EMGdi Envelopes Vs Onset and Offset Detectors

The automatic detection of onsets and offsets of EMGdi activity is crucial for evaluating the synchronization between the patient’s respiratory effort and the ventilator. The detection algorithm employed is consistent with those used in previous studies [11], [23] and is directly applied to the envelopes. This algorithm is based on the computation of a dynamically changing threshold cycle-by-cycle, determined by the most probable value calculated using kernel density estimation (KDE) method.

The algorithms were evaluated using MD to assess bias, SD to measure dispersion, and RMSE as a global metric combining both aspects. For onset bias, techniques using the first envelope exhibited the lowest MD, with similar bias values ranging between −119 ms and −130 ms, where fSE demonstrated the least bias. The application of a second moving average of 300 ms resulted in a substantial increase in bias across all techniques (ranging from −268 ms to −326 ms). Finally, upon employing the third envelope, the behavior of RMS-based and fSE-based envelopes diverged; the RMS-based detections advanced relative to the scorer detections (between 145 ms and 217 ms), while the fSE-based detections were delayed concerning the scorers, with a delay ranging between −193 ms and −196 ms compared to the detections from the first and second envelopes.

In terms of offset bias, it was generally considerably greater and of the opposite sign, exhibiting a similar pattern to the onset bias: the first envelope yielded the lowest bias (between 244 ms and 348 ms), while the second envelope increased the bias (between 421 ms and 475 ms). In this case, the third envelope exhibited clearly higher bias in RMS-based envelopes (between 428 ms and 518 ms) compared to the fSE-based envelopes (between 283 ms and 286 ms).

Since the onset and offset detection algorithm has inherent bias, a correction algorithm for this bias was proposed. This algorithm is based on estimating the I2BSNR of the EMGdi signal and applying a corrective equation derived from EMGdi signals simulated with varying levels of I2BSNR. The application of this offset correction algorithm substantially reduces bias in the detection of both onsets and offsets across all techniques, except for the onsets calculated with the third envelope of the RMS-based techniques. Excluding these three cases, the bias values ranged from −28 ms to −122 ms, with fSE-based techniques achieving the lowest onset bias (−30 ms for the fSE technique applied to the original signal and −28 ms when adaptive filtering is applied first). This exception suggests that the current I2BSNR-based correction model is not well suited to RMS-e3 onsets, whereas it behaves consistently for the fSE-based envelopes. Regarding offset bias, the I2BSNR-based correction technique successfully reduces bias in all cases, with fSE-based techniques again yielding the lowest offset bias (115 ms for the fSE technique applied to the original signal and 120 ms when adaptive filtering is applied first).

As expected, the bias correction algorithm based on I2BSNR has minimal effect on the dispersion of onset and offset detectors. For onset SD, values are quite comparable among the different techniques (ranging from 256 ms to 312 ms), except for the three cases involving the third RMS envelope, with the technique utilizing adaptive filtering together with gating exhibiting the lowest SD. The SD for offsets is clearly greater than that for onsets, with fSE-based techniques achieving the lowest offset SD (332 ms for the fSE technique applied to the original signal and 315 ms when adaptive filtering is applied first).

It should be noted that there is no universally accepted physiological definition of the “true” end of inspiratory muscle activity in surface EMGdi. Different groups have used, for example, a fixed percentage of the peak amplitude (often 70% of the peak sEMG envelope) or a return to baseline criterion to define the end of inspiration in studies of respiratory muscle activity and patient-ventilator interaction [8], [21], [23], [32], [33]. In this study, the reference offset was defined by the expert clinicians as the end of the main EMGdi burst when the activity returned to basal expiratory levels, consistent with their established practice for quantifying neural inspiratory time and cycling asynchronies in NIV. The automatic offset detectors were calibrated and evaluated against this clinically used definition.

Overall, when considering both the corrected bias and dispersion, the three techniques with the lowest RMSE onsets are (1) adaptive filtering with gating using the first envelope (265 ms), (2) fSE applied to the original signal (270 ms), and (3) fSE applied with adaptive filtering (271 ms). However, the adaptive filtering with gating configuration attains its onset RMSE on a smaller subset of inspiratory cycles, so fewer cycles contribute to the timing statistics. For offsets, the three techniques with the lowest RMSE are (1) fSE applied with adaptive filtering (315 ms), (2) fSE applied to the original signal (332 ms), and adaptive filtering with gating using the first envelope (404 ms). For comparison, the RMSD between human scorers is 71.4 ms for onsets and 76.1 ms for offsets, after excluding all cycles with a difference greater than 150 ms, which represented about 30% of the total.

A technical limitation of this study is that EMGdi was acquired in the original clinical trial with analogue pre-filtering between 0 and 200 Hz at a sampling rate of 500 Hz, which precludes analysis of higher-frequency EMG components. However, the proposed pipeline focuses on inspiratory event detection and RMS-/fSE-based indices within the 5-200 Hz band rather than on high-frequency spectral or conduction velocity analysis. For these applications, we therefore do not expect these acquisition settings to materially affect the reported performance.

By automatically detecting each inspiratory cycle and its onset and offset in the EMGdi signal, the proposed methodology enables a detailed breath-by-breath analysis of PVA. This is particularly valuable given the low-SNR and artefact-prone nature of overnight NIV recordings, which further complicate surface EMGdi analysis. This reliable offline quantification of neural respiratory timing in overnight home NIV recordings gives clinicians objective metrics to refine trigger and cycling settings on a night-to-night basis, thereby reducing PVA burden and supporting better long-term outcomes in COPD. Beyond cycle-level performance, the integration of fSE envelopes with a bias correction driven by signal quality directly addresses two major limitations of previous approaches: the susceptibility to ECG artefacts and the presence of systematic onset/offset shifts. This reinforces the potential of the method not only as a research tool but also as a clinically actionable framework for synchrony monitoring.

## VI. Conclusion

This study introduces and validates an automated pipeline for detecting all respiratory EMGdi cycles and their onset/offset instants in overnight home NIV recordings from severe COPD patients, where the EMGdi activity is extremely weak, often contaminated by ECG artefacts, making its detection particularly challenging. By systematically comparing ECG suppression strategies and RMS- and fSE-based envelopes, we show that fSE envelopes, particularly when combined with adaptive filtering, provide a favorable balance between event detection performance and onset/offset timing errors that remain within clinically acceptable limits for patient-ventilator interaction assessment.

Overnight home NIV recordings often provide surface EMGdi signals that are difficult to interpret consistently, which underscores the need for automated analysis approaches that can reliably extract respiratory timing information. By addressing this challenge, the proposed pipeline enables reliable breath-by-breath extraction of EMGdi activity and its onset-offset markers, providing clinicians with objective overnight metrics to refine trigger and cycling thresholds on successive nights, offering a practical route to lessen PVA burden and enhance long-term NIV efficacy in severe COPD. Compared to prior approaches relying mainly on RMS-based envelopes or semi-automatic marking, this is, to our knowledge, the first fully automated pipeline that combines fSE envelopes with a bias correction based on signal quality. This combination proved superior in both detection accuracy and bias control, directly addressing the main barriers that limited clinical usability of EMGdi analysis in home NIV. Although the present validation was limited to a single-center cohort, the methodology is readily portable to larger, multi-night datasets.

Future work should extend external validation to larger, multi-night and multicenter cohorts and test whether continuous overnight use of this tool can guide individualized NIV adjustments, potentially reducing ineffective efforts and delayed cycling in daily practice. In parallel, developing lightweight implementations suitable for bedside and home use and integrating them into portable diaphragmatic monitoring systems [34] will be key to facilitating translation of this approach into clinical practice.

## Supplementary Materials

Supplementary Materials

## References

[1] Global Initiative for Chronic Obstructive Lung Disease-GOLD. 2024 GOLD Report. Accessed: Nov. 13, 2024. [Online]. Available: https://goldcopd.org/2024-gold-report/

[2] D. Poddighe , “Respiratory muscle dysfunction in acute and chronic respiratory failure: How to diagnose and how to treat?” Eur. Respiratory Rev., vol. 33, no. 174, Dec. 2024, Art. no. 240150, doi: 10.1183/16000617.0150-2024. PMC11615664 39631928

[3] C. R. Osadnik, V. S. Tee, K. V. Carson-Chahhoud, J. Picot, J. A. Wedzicha, and B. J. Smith, “Non-invasive ventilation for the management of acute hypercapnic respiratory failure due to exacerbation of chronic obstructive pulmonary disease,” Cochrane Database Systematic Rev., vol. 2017, no. 7, Jul. 2017, Art. no. 004104, doi: 10.1002/14651858.cd004104.pub4. PMC6483555 28702957

[4] M. L. Duiverman, A. S. Huberts, L. A. van Eykern, G. Bladder, and P. J. Wijkstra, “Respiratory muscle activity and patient–ventilator asynchrony during different settings of noninvasive ventilation in stable hypercapnic COPD: Does high inspiratory pressure lead to respiratory muscle unloading?” Int. J. Chronic Obstructive Pulmonary Disease, vol. Volume 12, pp. 243–257, Jan. 2017, doi: 10.2147/copd.s119959. PMC5238808 28138234

[5] T. Raveling , “Home noninvasive ventilation in severe COPD: In whom does it work and how?” ERJ Open Res., vol. 10, no. 1, pp. 00600–02023, Jan. 2024, doi: 10.1183/23120541.00600-2023. 38348241 PMC10860207

[6] L. Vignaux , “Patient–ventilator asynchrony during non-invasive ventilation for acute respiratory failure: A multicenter study,” Intensive Care Med., vol. 35, no. 5, pp. 840–846, May 2009, doi: 10.1007/s00134-009-1416-5. 19183949

[7] American Thoracic Society (ATS) / European Respiratory Society (ERS), “ATS/ERS statement on respiratory muscle testing,” Am. J. Respir. Crit. Care Med., vol. 166, no. 4, pp. 518–624, Aug. 2002, doi: 10.1164/rccm.166.4.518. 12186831

[8] A. H. Jonkman , “Analysis and applications of respiratory surface EMG: Report of a round table meeting,” Crit. Care, vol. 28, no. 1, p. 2, Jan. 2024, doi: 10.1186/s13054-023-04779-x. 38166968 PMC10759550

[9] A. Oltmann, J. Graßhoff, N. Lange, T. Knopp, and P. Rostalski, “A quantitative comparison of electrode positions for respiratory surface EMG,” IEEE Trans. Biomed. Eng., vol. 72, no. 8, pp. 2437–2446, Aug. 2025, doi: 10.1109/TBME.2025.3543644. 40036440

[10] F. Mojoli , “Timing of inspiratory muscle activity detected from airway pressure and flow during pressure support ventilation: The waveform method,” Crit. Care, vol. 26, no. 1, p. 32, Jan. 2022, doi: 10.1186/s13054-022-03895-4. 35094707 PMC8802480

[11] L. Sarlabous , “Electromyography-based respiratory onset detection in COPD patients on non-invasive mechanical ventilation,” Entropy, vol. 21, no. 3, p. 258, Mar. 2019, doi: 10.3390/e21030258. 33266973 PMC7514739

[12] J. Sauer, J. Graßhoff, N. M. Carbon, W. M. Koch, S. Weber-Carstens, and P. Rostalski, “Automated characterization of patient–ventilator interaction using surface electromyography,” Ann. Intensive Care, vol. 14, no. 1, p. 32, Feb. 2024, doi: 10.1186/s13613-024-01259-5. 38407643 PMC10897101

[13] E. Petersen, J. Sauer, J. Graßhoff, and P. Rostalski, “Removing cardiac artifacts from single-channel respiratory electromyograms,” IEEE Access, vol. 8, pp. 30905–30917, 2020, doi: 10.1109/ACCESS.2020.2972731.

[14] R. W. van Leuteren, G. J. Hutten, C. G. de Waal, P. Dixon, A. H. van Kaam, and F. H. de Jongh, “Processing transcutaneous electromyography measurements of respiratory muscles, a review of analysis techniques,” J. Electromyogr. Kinesiol., vol. 48, pp. 176–186, Oct. 2019, doi: 10.1016/j.jelekin.2019.07.014. 31401341

[15] R. S. P. Warnaar, A. D. Cornet, A. Beishuizen, C. M. Moore, D. W. Donker, and E. Oppersma, “Advanced waveform analysis of diaphragm surface EMG allows for continuous non-invasive assessment of respiratory effort in critically ill patients at different PEEP levels,” Crit. Care, vol. 28, no. 1, p. 195, Jun. 2024, doi: 10.1186/s13054-024-04978-0. 38851709 PMC11162564

[16] C. Zhan, L. F. Yeung, and Z. Yang, “A wavelet-based adaptive filter for removing ECG interference in EMGdi signals,” J. Electromyogr. Kinesiol., vol. 20, no. 3, pp. 542–549, Jun. 2010, doi: 10.1016/j.jelekin.2009.07.007. 19692270

[17] A. H. Jonkman, R. Juffermans, J. Doorduin, L. M. A. Heunks, and J. Harlaar, “Estimated ECG subtraction method for removing ECG artifacts in esophageal recordings of diaphragm EMG,” Biomed. Signal Process. Control, vol. 69, Aug. 2021, Art. no. 102861, doi: 10.1016/j.bspc.2021.102861.

[18] G. Lu , “Removing ECG noise from surface EMG signals using adaptive filtering,” Neurosci. Lett., vol. 462, no. 1, pp. 14–19, Sep. 2009, doi: 10.1016/j.neulet.2009.06.063. 19559751

[19] A. Torres, J. A. Fiz, and R. Jan, “Cancellation of cardiac interference in diaphragm EMG signals using an estimate of ECG reference signal,” in Proc. Medit. Conf. Med. Biol. Eng. Comput., Jan. 2014, pp. 1000–1004, doi: 10.1007/978-3-319-00846-2_248.

[20] S. Dacha , “Comparison between manual and (Semi-)Automated analyses of esophageal diaphragm electromyography during endurance cycling in patients with COPD,” Frontiers Physiol., vol. 10, p. 885, Jul. 2019, doi: 10.3389/fphys.2019.00885. PMC6637315 31354525

[21] A. Rodrigues , “Semi-automated detection of the timing of respiratory muscle activity: Validation and first application,” Frontiers Physiol., vol. 12, Jan. 2022, Art. no. 794598, doi: 10.3389/fphys.2021.794598. PMC8762204 35046839

[22] L. Estrada, A. Torres, L. Sarlabous, and R. Jané, “Improvement in neural respiratory drive estimation from diaphragm electromyographic signals using fixed sample entropy,” IEEE J. Biomed. Health Informat., vol. 20, no. 2, pp. 476–485, Mar. 2016, doi: 10.1109/JBHI.2015.2398934. 25667362

[23] L. Estrada, A. Torres, L. Sarlabous, and R. Jané, “Onset and offset estimation of the neural inspiratory time in surface diaphragm electromyography: A pilot study in healthy subjects,” IEEE J. Biomed. Health Informat., vol. 22, no. 1, pp. 67–76, Jan. 2018, doi: 10.1109/JBHI.2017.2672800. 28237936

[24] A. Tlimat , “Artificial intelligence for the detection of patient–ventilator asynchrony,” Respiratory Care, vol. 70, no. 5, pp. 583–592, May 2025, doi: 10.1089/respcare.12540. 40178919 PMC12369843

[25] H. F. R. Prechtl, L. A. Van Eykern, and M. J. O'Brien, “Respiratory muscle EMG in newborns: A non-intrusive method,” Early Human Develop., vol. 1, no. 3, pp. 265–283, Dec. 1977, doi: 10.1016/0378-3782(77)90040-8. 617314

[26] J. Pan and W. J. Tompkins, “A real-time QRS detection algorithm,” IEEE Trans. Biomed. Eng., vols. BME–32, no. 3, pp. 230–236, Mar. 1985, doi: 10.1109/TBME.1985.325532. 3997178

[27] L. Estrada, A. Torres, L. Sarlabous, and R. Jané, “Influence of parameter selection in fixed sample entropy of surface diaphragm electromyography for estimating respiratory activity,” Entropy, vol. 19, no. 9, p. 460, Sep. 2017, doi: 10.3390/e19090460.

[28] M. Lozano-García , “Noninvasive assessment of inspiratory muscle neuromechanical coupling during inspiratory threshold loading,” IEEE Access, vol. 7, pp. 183634–183646, 2019, doi: 10.1109/ACCESS.2019.2960077.

[29] M. Lozano-García , “Noninvasive assessment of neuromechanical and neuroventilatory coupling in COPD,” IEEE J. Biomed. Health Informat., vol. 26, no. 7, pp. 3385–3396, Jul. 2022, doi: 10.1109/JBHI.2022.3166255. 35404825

[30] M. Lozano-García , “Noninvasive assessment of neuromechanical coupling and mechanical efficiency of parasternal intercostal muscle during inspiratory threshold loading,” Sensors, vol. 21, no. 5, p. 1781, Mar. 2021, doi: 10.3390/s21051781. 33806463 PMC7961675

[31] A. A. Koopman, J. van Dijk, E. Oppersma, R. G. T. Blokpoel, and M. C. J. Kneyber, “Surface electromyography to quantify neuro-respiratory drive and neuro-mechanical coupling in mechanically ventilated children,” Respiratory Res., vol. 24, no. 1, p. 77, Mar. 2023, doi: 10.1186/s12931-023-02374-w. PMC10010013 36915106

[32] J. Doorduin, C. A. Sinderby, J. Beck, J. G. van der Hoeven, and L. M. Heunks, “Automated patient-ventilator interaction analysis during neurally adjusted non-invasive ventilation and pressure support ventilation in chronic obstructive pulmonary disease,” Crit. Care, vol. 18, no. 5, p. 550, Oct. 2014, doi: 10.1186/s13054-014-0550-9. 25307894 PMC4207887

[33] L. Liu , “Automatic adjustment of the inspiratory trigger and cycling-off criteria improved patient-ventilator asynchrony during pressure support ventilation,” Frontiers Med., vol. 8, Nov. 2021, Art. no. 752508, doi: 10.3389/fmed.2021.752508. PMC8632800 34869448

[34] J. Curley , “Feasibility analysis of a portable diaphragmatic efficiency monitor for CSCI patients,” IEEE J. Translational Eng. Health Med., vol. 13, pp. 246–250, 2025, doi: 10.1109/JTEHM.2025.3574553. PMC12310168 40740834

