# Supplementary material for: Automatic Detection of Onset and Offset of Respiratory Electromyographic Activity in Severe COPD Patients on Non-Invasive Mechanical Ventilation
Source: IEEE J Transl Eng Health Med. 2026 Jan 23;14:55–66. doi: 10.1109/JTEHM.2026.3657639 (PMC12947965; doi:10.1109/JTEHM.2026.3657639)
Supplement: Supplementary Materials [file supp1-3657639.pptx]

## Slide 1
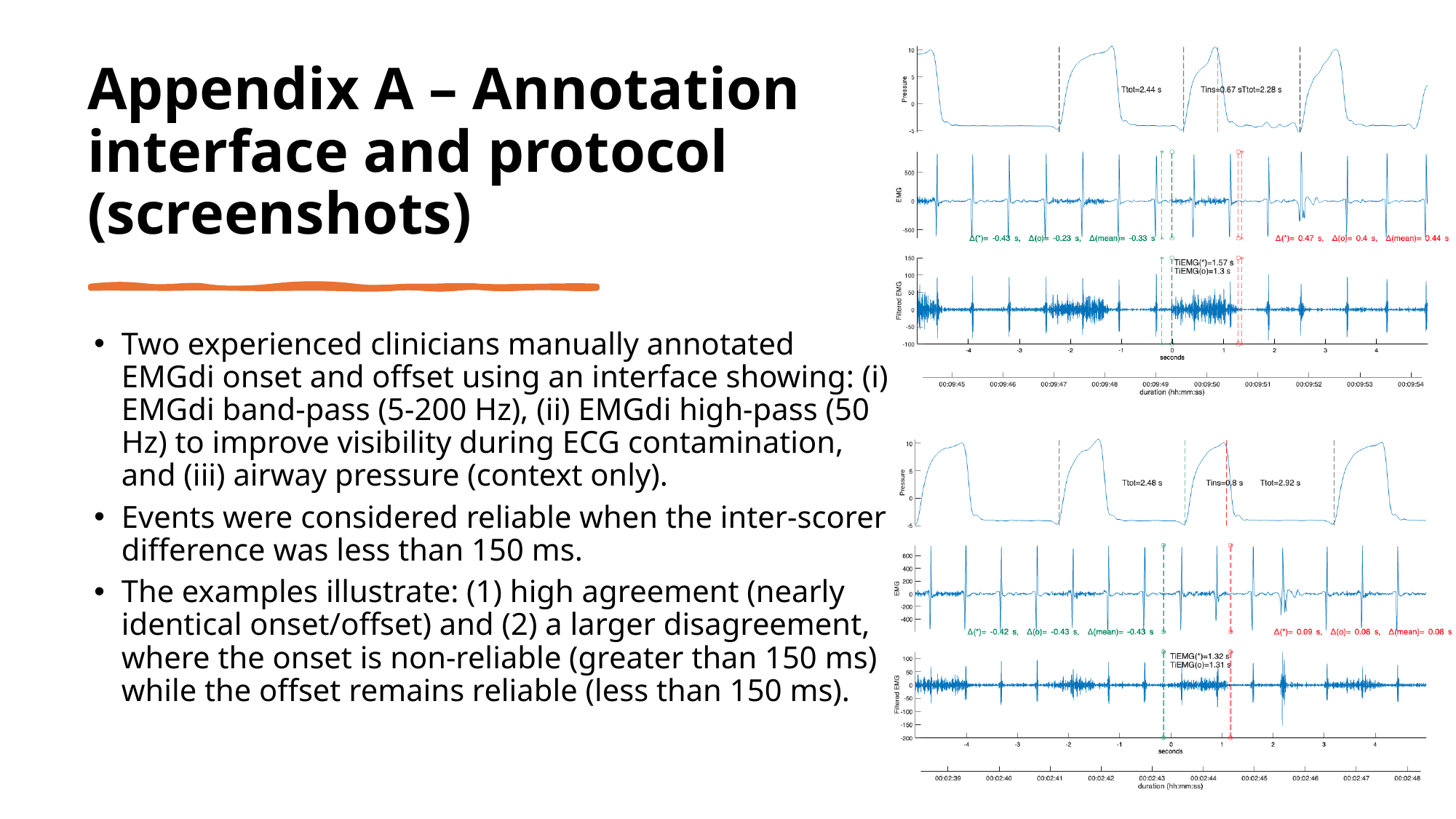

Appendix A – Annotation interface and protocol (screenshots)
Two experienced clinicians manually annotated EMGdi onset and offset using an interface showing: (i) EMGdi band-pass (5-200 Hz), (ii) EMGdi high-pass (50 Hz) to improve visibility during ECG contamination, and (iii) airway pressure (context only).
Events were considered reliable when the inter-scorer difference was less than 150 ms.
The examples illustrate: (1) high agreement (nearly identical onset/offset) and (2) a larger disagreement, where the onset is non-reliable (greater than 150 ms) while the offset remains reliable (less than 150 ms).

## Slide 2
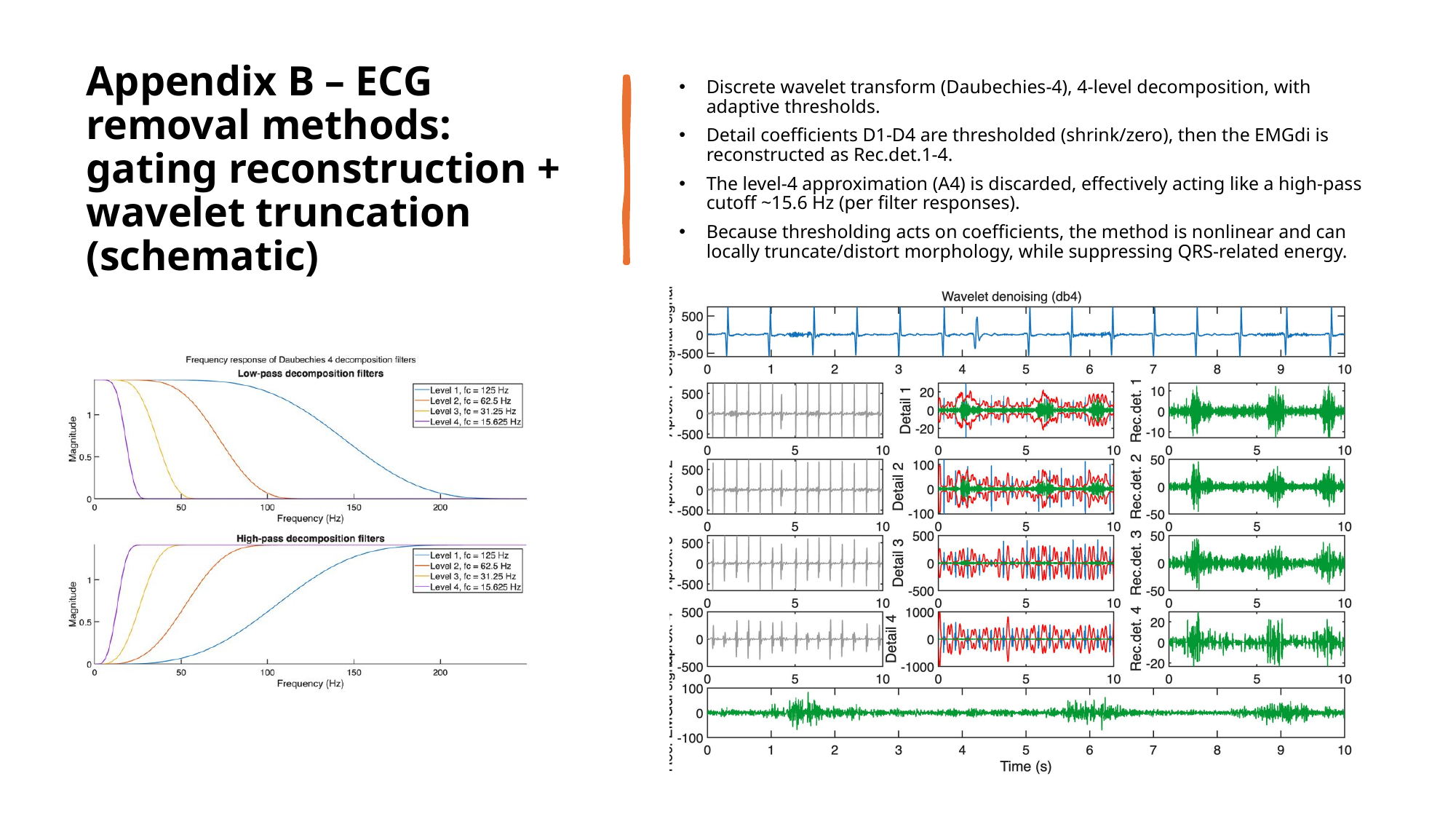

Appendix B – ECG removal methods: gating reconstruction + wavelet truncation (schematic)
Discrete wavelet transform (Daubechies-4), 4-level decomposition, with adaptive thresholds.
Detail coefficients D1-D4 are thresholded (shrink/zero), then the EMGdi is reconstructed as Rec.det.1-4.
The level-4 approximation (A4) is discarded, effectively acting like a high-pass cutoff ~15.6 Hz (per filter responses).
Because thresholding acts on coefficients, the method is nonlinear and can locally truncate/distort morphology, while suppressing QRS-related energy.

## Slide 3
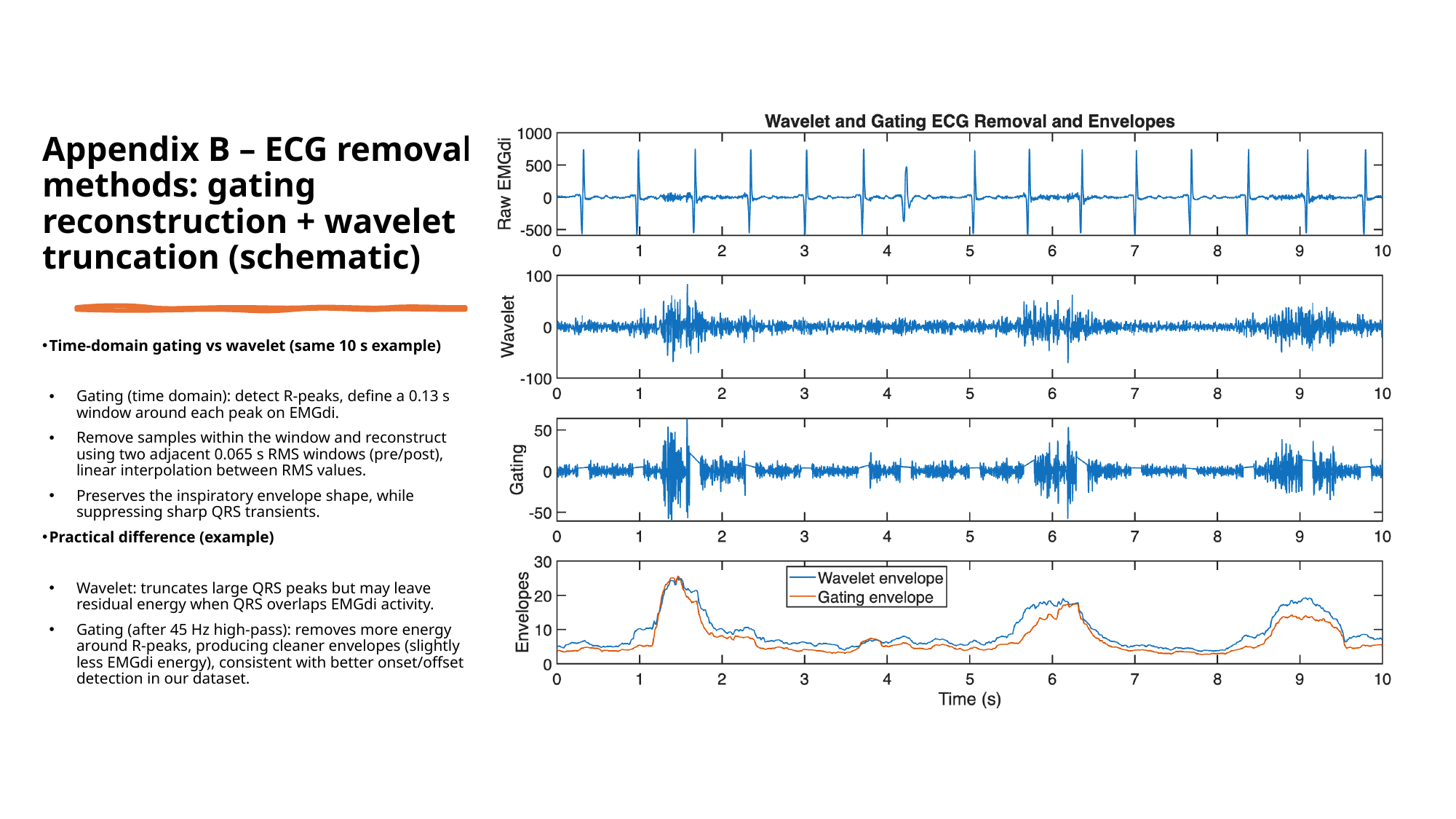

Appendix B – ECG removal methods: gating reconstruction + wavelet truncation (schematic)
Time-domain gating vs wavelet (same 10 s example)
Gating (time domain): detect R-peaks, define a 0.13 s window around each peak on EMGdi.
Remove samples within the window and reconstruct using two adjacent 0.065 s RMS windows (pre/post), linear interpolation between RMS values.
Preserves the inspiratory envelope shape, while suppressing sharp QRS transients.
Practical difference (example)
Wavelet: truncates large QRS peaks but may leave residual energy when QRS overlaps EMGdi activity.
Gating (after 45 Hz high-pass): removes more energy around R-peaks, producing cleaner envelopes (slightly less EMGdi energy), consistent with better onset/offset detection in our dataset.

## Slide 4
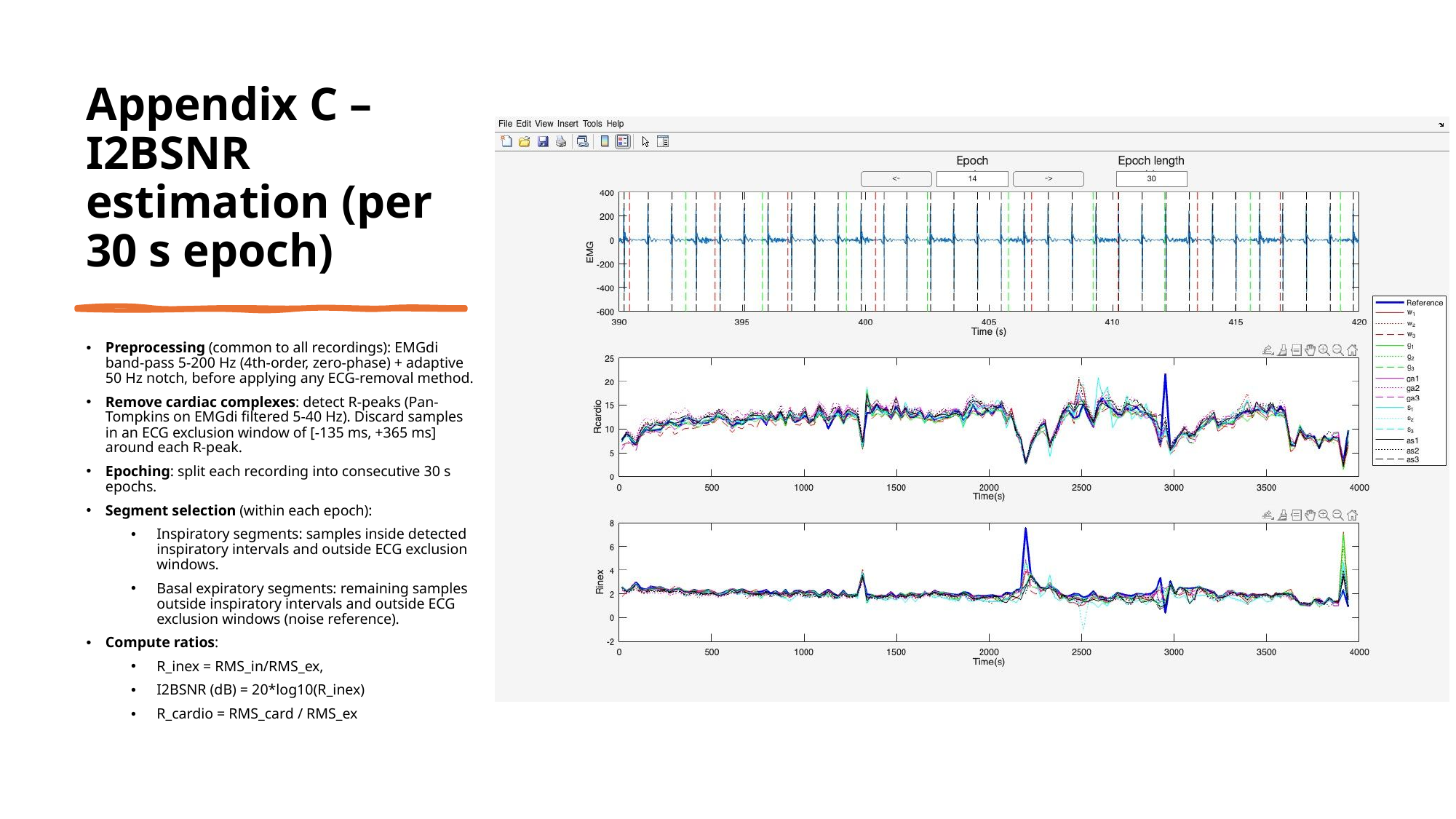

Appendix C – I2BSNR estimation (per 30 s epoch)
Preprocessing (common to all recordings): EMGdi band-pass 5-200 Hz (4th-order, zero-phase) + adaptive 50 Hz notch, before applying any ECG-removal method.
Remove cardiac complexes: detect R-peaks (Pan-Tompkins on EMGdi filtered 5-40 Hz). Discard samples in an ECG exclusion window of [-135 ms, +365 ms] around each R-peak.
Epoching: split each recording into consecutive 30 s epochs.
Segment selection (within each epoch):
Inspiratory segments: samples inside detected inspiratory intervals and outside ECG exclusion windows.
Basal expiratory segments: remaining samples outside inspiratory intervals and outside ECG exclusion windows (noise reference).
Compute ratios:
R_inex = RMS_in/RMS_ex,
I2BSNR (dB) = 20*log10(R_inex)
R_cardio = RMS_card / RMS_ex

## Slide 5
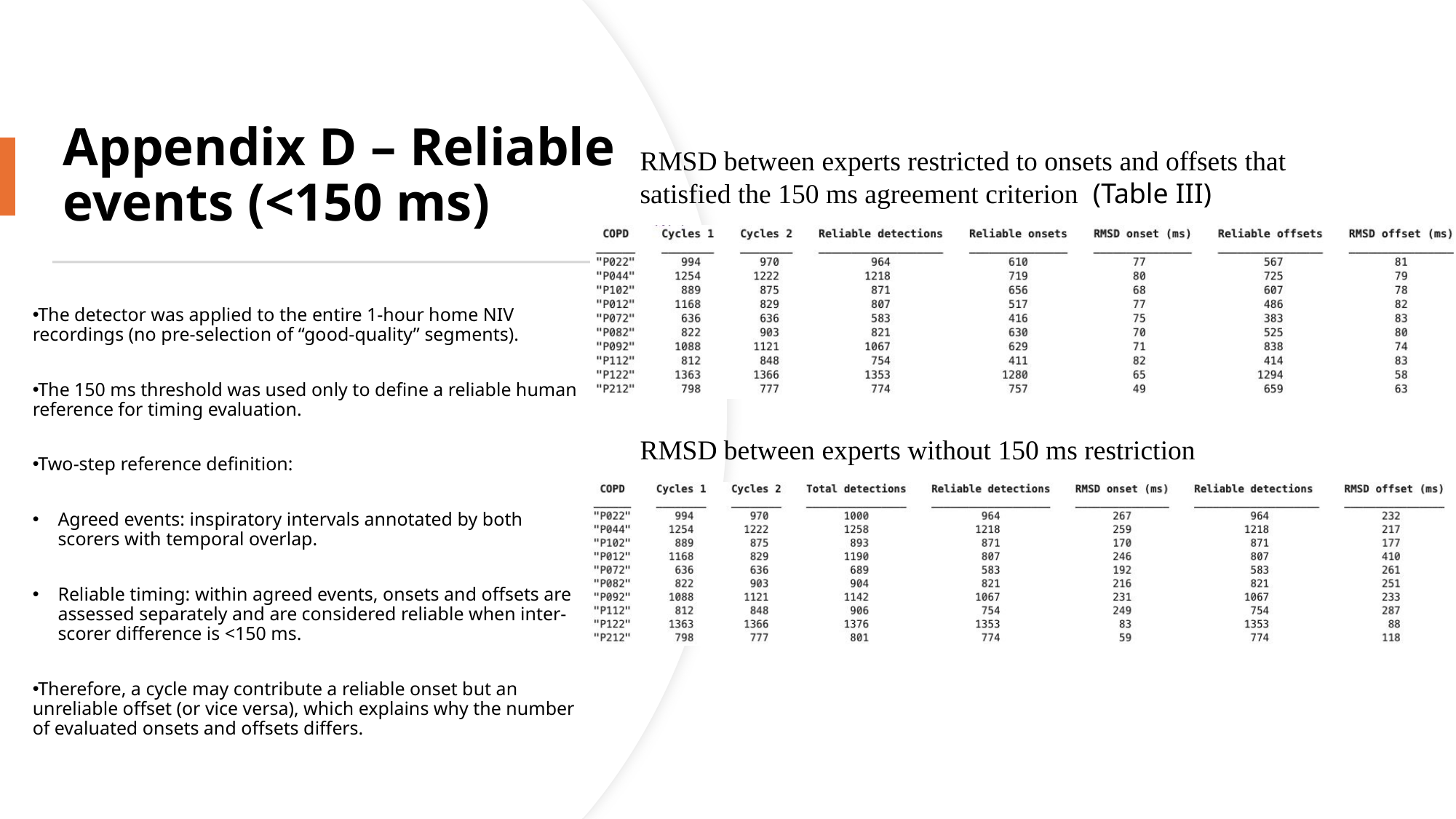

Appendix D – Reliable events (<150 ms)
RMSD between experts restricted to onsets and offsets that satisfied the 150 ms agreement criterion (Table III)
The detector was applied to the entire 1-hour home NIV recordings (no pre-selection of “good-quality” segments).
The 150 ms threshold was used only to define a reliable human reference for timing evaluation.
Two-step reference definition:
Agreed events: inspiratory intervals annotated by both scorers with temporal overlap.
Reliable timing: within agreed events, onsets and offsets are assessed separately and are considered reliable when inter-scorer difference is <150 ms.
Therefore, a cycle may contribute a reliable onset but an unreliable offset (or vice versa), which explains why the number of evaluated onsets and offsets differs.
RMSD between experts without 150 ms restriction

## Slide 6
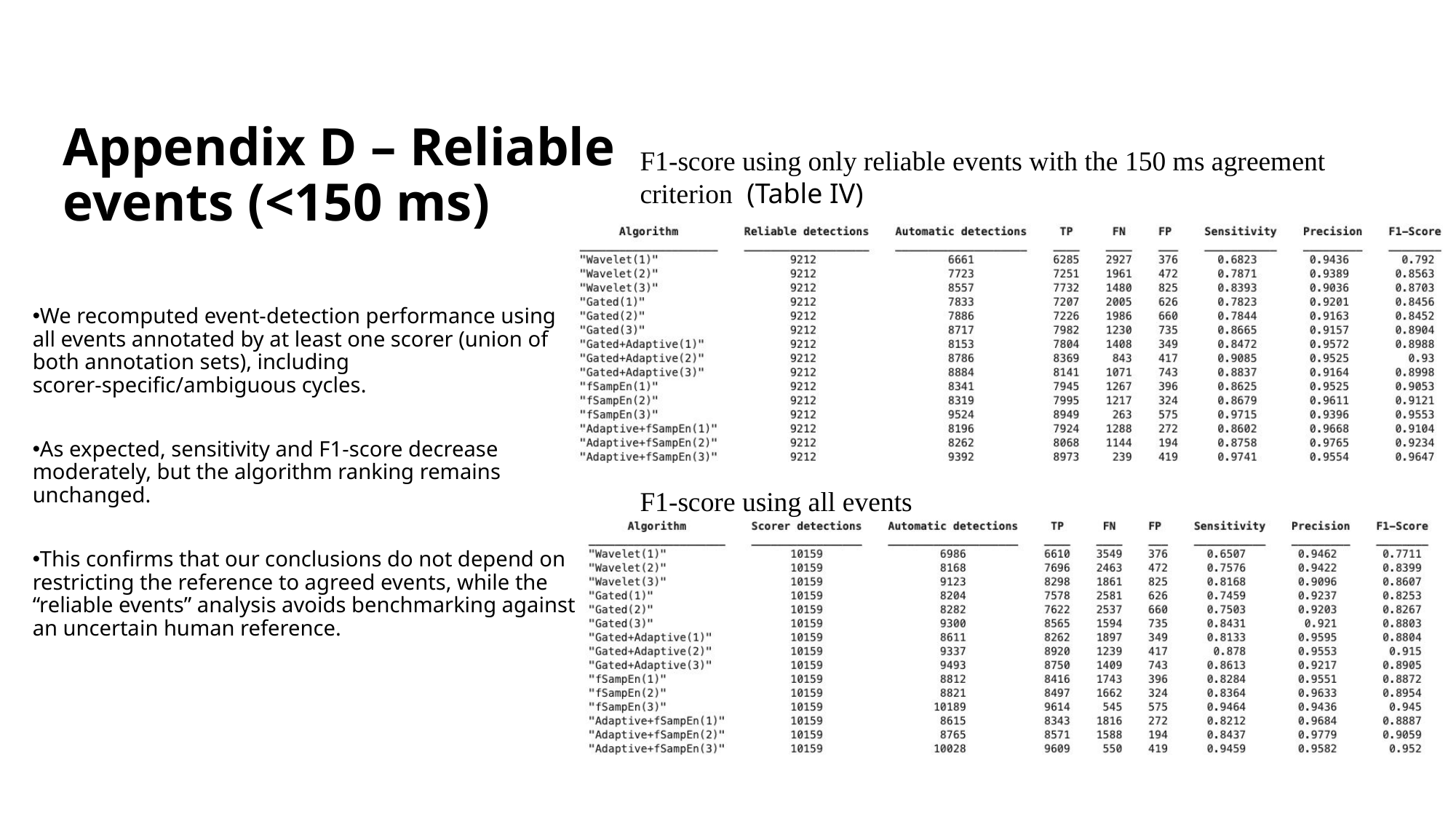

Appendix D – Reliable events (<150 ms)
F1-score using only reliable events with the 150 ms agreement criterion (Table IV)
We recomputed event-detection performance using all events annotated by at least one scorer (union of both annotation sets), including scorer-specific/ambiguous cycles.
As expected, sensitivity and F1-score decrease moderately, but the algorithm ranking remains unchanged.
This confirms that our conclusions do not depend on restricting the reference to agreed events, while the “reliable events” analysis avoids benchmarking against an uncertain human reference.
F1-score using all events

## Slide 7
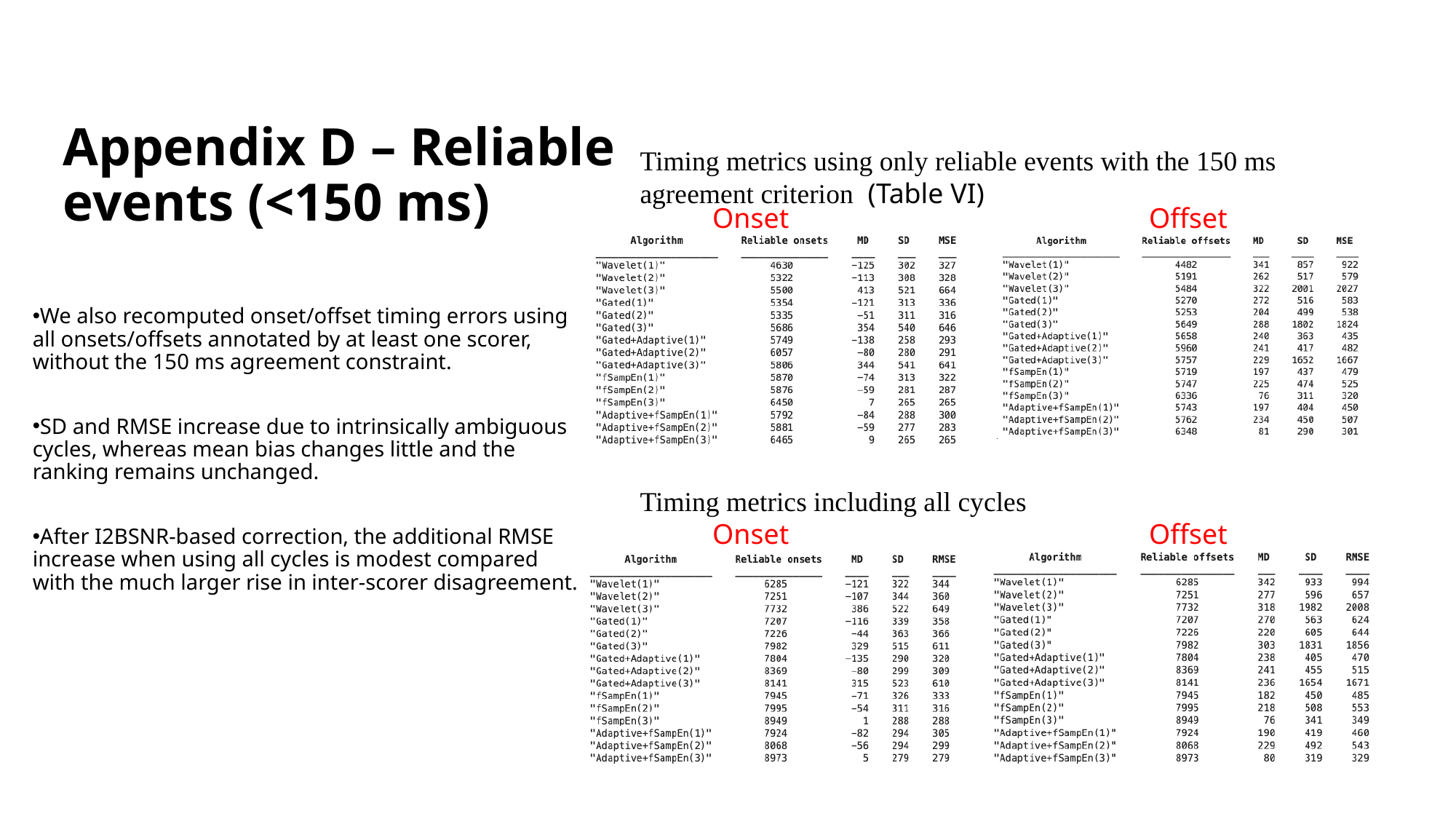

Appendix D – Reliable events (<150 ms)
Timing metrics using only reliable events with the 150 ms agreement criterion (Table VI)
Onset				Offset
We also recomputed onset/offset timing errors using all onsets/offsets annotated by at least one scorer, without the 150 ms agreement constraint.
SD and RMSE increase due to intrinsically ambiguous cycles, whereas mean bias changes little and the ranking remains unchanged.
After I2BSNR-based correction, the additional RMSE increase when using all cycles is modest compared with the much larger rise in inter-scorer disagreement.
Timing metrics including all cycles
Onset				Offset
